# Paraneoplastic Hypereosinophilia in Poorly Differentiated Adenocarcinoma of the Lung

**DOI:** 10.7759/cureus.34386

**Published:** 2023-01-30

**Authors:** Sarah Ream, Piruthiviraj Natarajan, Shruti Gupta, Elsa Sotelo-Rafiq, Dan Schuller

**Affiliations:** 1 Department of Internal Medicine, Texas Tech University Health Sciences Center El Paso, Paul L. Foster School of Medicine, El Paso, USA; 2 Department of Internal Medicine, The Hospitals of Providence- Transmountain/Texas Tech University Health Sciences Center El Paso, Paul L. Foster School of Medicine, El Paso, USA; 3 Department of Pathology and Laboratory Medicine, Las Palmas Medical Center, El Paso, USA; 4 Department of Internal Medicine/Pulmonary and Critical Care Medicine, The Hospitals of Providence- Transmountain/Texas Tech University Health Sciences Center El Paso, Paul L. Foster School of Medicine, El Paso, USA

**Keywords:** hypereosinophilia, lung adenocarcinoma, poorly differentiated lung adenocarcinoma, paraneoplastic hypereosinophilia, paraneoplastic syndrome

## Abstract

It is well-documented that lung and bronchus cancers are the leading cause of cancer death in the United States in both male and female patients, with lung adenocarcinoma accounting for the highest prevalence of lung cancers. Significant eosinophilia in the setting of lung adenocarcinoma has been documented in a few reports, being described as a rare paraneoplastic syndrome. We report on an 81-year-old female with hypereosinophilia-associated lung adenocarcinoma. A chest film showed a right lung mass, which was not apparent on a chest film 1 year prior, in the setting of significant leukocytosis of 27.90 x 10^3^/mm^3^ with eosinophilia of 6.40 x 10^3^/mm^3^. A computed tomography (CT) chest, obtained during admission, demonstrated significant right lower lobe mass enlargement since the previous study completed 5 months prior, with new occlusion of bronchi and pulmonary vessels to the region of the mass. Our observations are consistent with prior reports showing that the presence of eosinophilia in lung cancers may indicate rapid disease progression.

## Introduction

It is well-documented that lung and bronchus cancers are the leading cause of cancer death in the United States in both male and female patients, and lung adenocarcinoma accounts for the highest prevalence of lung cancers [1,2]. Though most often seen in hematologic malignancies, significant eosinophilia in the setting of lung adenocarcinoma has been described as a rare paraneoplastic syndrome [3-5]. The presence of this paraneoplastic phenomenon, with increased serum eosinophils, has been recognized as a poor prognostic feature reflecting advanced disease and dissemination with a more rapid disease progression than expected [2-5]. We report on an 81-year-old female with hypereosinophilia associated with poorly differentiated lung adenocarcinoma.

This article was previously presented as a meeting abstract at the 2022 TXACP Annual Meeting on October 29, 2022.

## Case presentation

**Figure 1:**
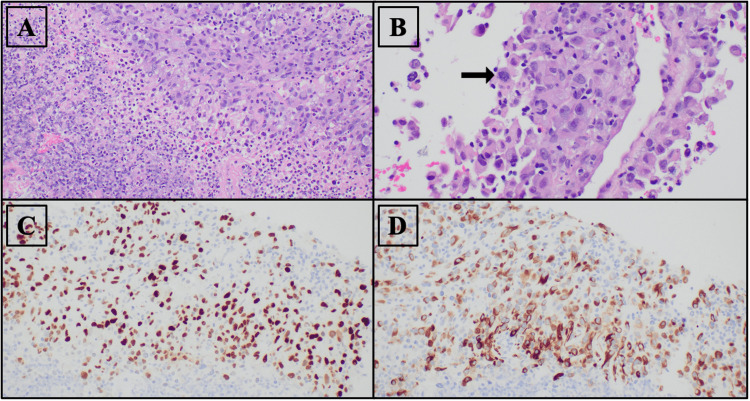
A: Upper right of image demonstrates tumor and lower left shows extensive inflammation. B: Demonstrating a higher magnification of the image in pane A, showing malignant features of non-small cell carcinoma; large pleomorphic cells with prominent nucleoli are seen. A large mitotic figure is shown (black arrow). C: Thyroid transcription factor-1 (TTF-1) immunohistochemical stain for lung adenocarcinoma, staining nuclei. D: Cytokeratin 7 (CK7) immunohistochemical stain for lung adenocarcinoma, staining cytoplasm.

We report on an 81-year-old female, lifetime smoker, with a past medical history of newly diagnosed poorly differentiated lung adenocarcinoma with suspected metastasis, diabetes mellitus type II, hypertension, and obstructive sleep apnea. The patient initially presented with a reduced appetite and rapid weight loss; these symptoms with the patient’s history of smoking prompted imaging studies to be obtained. The patient was found to have a right lung mass on a CT chest, and 52 days later a CT-guided needle core biopsy diagnosed poorly differentiated, invasive lung adenocarcinoma with extensive necrosis (Figure 1).

**Figure 2:**
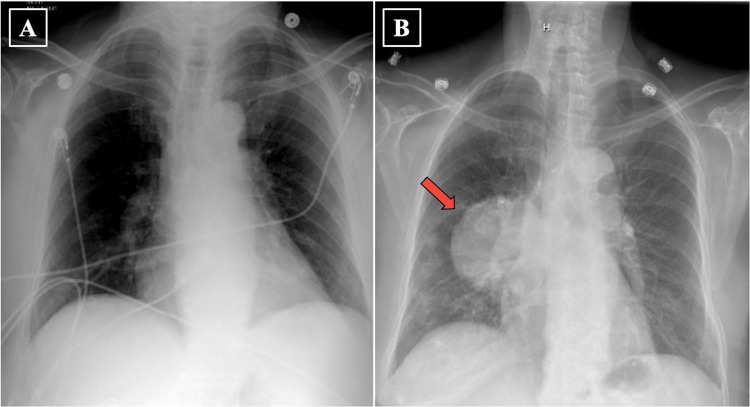
A: Chest film from 363 days prior to hospital admission. B: Hospital chest film showing a large superior segment right lower lobe mass (red arrow).

Three weeks after diagnosis, the patient presented to the hospital for a scheduled left adrenal gland mass biopsy that was canceled due to preoperative laboratory results showing a leukocytosis of 27.90 x 10^3^/mm^3^ with eosinophilia of 6.40 x 10^3^/mm^3^. The patient was admitted for further workup which was unremarkable for occult infection at that time. Compared to a negative chest film from 1 year prior (Figure 2A), a hospital chest film showed a right lower lobe mass (Figure 2B).

**Figure 3:**
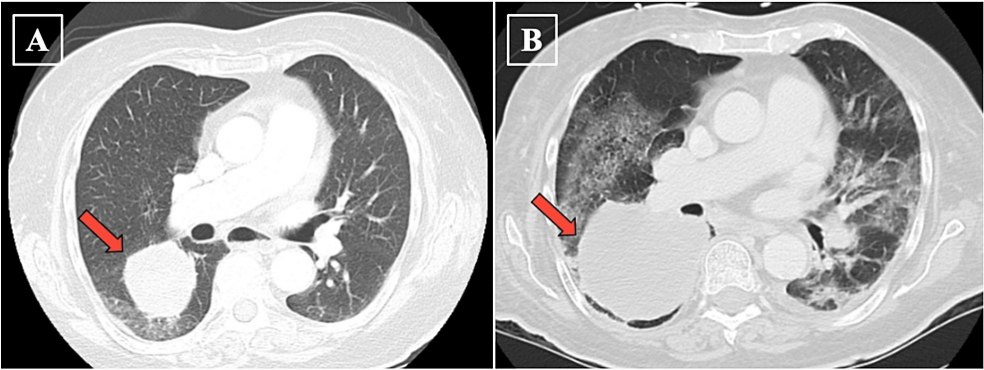
A: CT chest from 132 days prior to hospital admission CT chest (seen in pane B), demonstrating a right lower lobe lung mass (red arrow). B: CT chest obtained on day 9 of hospital admission demonstrating a larger right lower lobe lung mass (red arrow).

On hospital day 5, the patient developed a cough and shortness of breath, showing new lung infiltrates on a repeat chest film and empiric antimicrobials for nosocomial pneumonia were started; ongoing antibiotic therapy showed no response of leukocytosis pattern. On admission day 7 the patient became hypotensive, with a blood pressure measured at 90/60 mmHg, with increased difficulty breathing. After receiving a liter bolus of 0.9% normal saline she remained hypotensive with an oxygen saturation (SpO_2_) of 86% on 4 L of oxygen via nasal cannula. Subsequently, the patient was transferred to the intensive care unit (ICU) with acute hypoxemic respiratory failure, requiring a high-flow nasal cannula (HFNC). Physical exam demonstrated an increased work of breathing with bilateral wheezing. The patient was treated with methylprednisolone 60 mg every 6 hours, resulting in complete resolution of the eosinophilia and clear breath sounds bilaterally. Compared to a CT chest (Figure 3A) from 132 days prior (showing a 5.1 x 4.7 x 1.3 cm mass), a new CT chest (Figure 3B) showed a mass measuring 6.9 x 6.2 x 1.9 cm in the right lower lobe with new occlusion of bronchi and pulmonary vessels to the region of the mass.

Lung abnormalities were seen on bronchoscopy, finding a nearly obstructing exophytic, friable mass proximally in the superior segment of the right lower lobe. An interleukin-5 (IL-5) level returned negative at <1.0 pg/mL. On admission day 11, ICU day 4, the patient experienced worsening respiratory status and a new chest film demonstrated worsening diffuse bilateral pneumonia and/or edema. Following unsuccessful high-flow oxygen therapy, she was intubated. After 8 days of intubation, the patient was successfully extubated on admission day 19, and ICU day 12. However, given the extent of her disease, poor functional status, and lack of therapeutic options, the family decided on home hospice care.

## Discussion

Two separate studies (Mackintosh et al., 2014 and Wilson et al., 2012) found that the median doubling time for lung adenocarcinoma is 249 days and 387 days, respectively, however, we observed a much more rapid rate of growth as seen radiographically [6,7]. Though these studies have identified that current smokers experience increased mass doubling times compared to non-smokers, our observation reflects an even quicker doubling time compared to that of current smokers. Paraneoplastic hypereosinophilia is likely reflective of extensive disease and dissemination, supporting its poor prognostic identification. Our observations are consistent with prior reports showing that the presence of eosinophilia may indicate rapid disease progression.

Eosinophilia may be caused by many conditions. For our patient, infectious diseases and allergic disorders were ruled out. However, there were no investigations for an underlying autoimmune disorder. A patient’s smoking status, irrespective of cancer status, has been associated with an elevated eosinophil count in addition to the white blood cell (WBC) count. One study reported only a slight WBC count mean decrease of 0.27 x 109/L in 1 year from baseline following smoking cessation [8]. Therefore, it is unlikely that our patient’s smoking exposure is significantly attributable to the development of hypereosinophilia given the marked leukocytosis and eosinophilia we observed, further supporting a paraneoplastic hypereosinophilia syndrome.

While the underlying mechanism of eosinophilia in patients with lung cancer is not completely understood, one theory is thought to be due to tumor-produced cytokines, most notably IL-5, leading to bone marrow stimulation [9]. While eosinophil-promoting IL-5 may play a role in certain tumor characteristics and disease progression, its significance in lung adenocarcinoma is still unknown. Our observation does not reflect IL-5-driven eosinophilia, prompting further questions regarding alternative pathogenesis. However, it is worth noting that in addition to IL-5, multiple other cytokines and mediators have been linked to the development of eosinophilia: interleukin-3 (IL-3), interleukin-13 (IL-13), interferon-gamma (IFN-g) and granulocyte/macrophage colony-stimulating factor (GM-CSF), platelet-derived growth factor (PDGF), and nerve growth factor (NGF) [8,10,11].

## Conclusions

There is no standard treatment for paraneoplastic hypereosinophilia and the true outcomes of eosinophilia treatment remain unclear. The need for increased awareness of paraneoplastic hypereosinophilia-associated lung adenocarcinoma is needed due to its correlation with greater disease progression and poorer prognosis. Additional studies are needed to elucidate the actual incidence, underlying risk factors, pathogenesis, and impact of paraneoplastic hypereosinophilia in patients with lung cancer and there is a need to explore novel, targeted therapies for improved patient outcomes.
